# Airborne optical imaging technology: a road map in CIOMP

**DOI:** 10.1038/s41377-025-01776-3

**Published:** 2025-03-10

**Authors:** Ping Jia, Dapeng Tian, Yutang Wang, Dong Yao, Rui Xu, Jianwei Sun, Hui Sun, Bao Zhang

**Affiliations:** State Key Laboratory of Dynamic Optical Imaging and Measurement, Changchun Institute of Optics, Fine Mechanics and Physics, Chinese Academy of Sciences, 130033 Changchun, China

**Keywords:** Imaging and sensing, Optoelectronic devices and components

## Abstract

Airborne optical imaging can flexibly obtain the intuitive information of the observed scene from the air, which plays an important role of modern optical remote sensing technology. Higher resolution, longer imaging distance, and broader coverage are the unwavering pursuits in this research field. Nevertheless, the imaging environment during aerial flights brings about multi-source dynamic interferences such as temperature, air pressure, and complex movements, which forms a serious contradiction with the requirements of precision and relative staticity in optical imaging. As the birthplace of Chinese optical industry, the Changchun Institute of Optics, Fine Mechanics and Physics (CIOMP) has conducted the research on airborne optical imaging for decades, resulting in rich innovative achievements, completed research conditions, and exploring a feasible development path. This article provides an overview of the innovative work of CIOMP in the field of airborne optical imaging, sorts out the milestone nodes, and predicts the future development direction of this discipline, with the aim of providing inspiration for related research.

## Introduction

Airborne optical imaging leverages the flexibility and mobility of aerial vehicle platforms, offering irreplaceable advantages in terms of imaging coverage, resolution, and observation efficiency, and can compensate for the deficiencies of ground-based and space-based imaging at the application level. However, the flight of aerial vehicles from the ground to the air also introduces many difficulties for the optical systems they carry: the motion of the optical imaging system relative to the scene, aerodynamic interference, changes in environmental temperature/air pressure, vibrations of the carrier aircraft, and constraints on the volume/weight/power consumption of the installed equipment, etc. As application requirements evolve toward long-distance, high-resolution, wide field-of-view, and large data volumes, dynamic optical imaging represented by airborne optical imaging has also formed a distinctive research direction and has been deeply studied.

During different periods of development, the technical characteristics of airborne optical imaging also vary, which is inseparable from the development of the carrier platform and changes in the working environment and imaging requirements. As early as 1903, before powered aircraft were widely used, humans began to use hot air balloons and rockets carrying optical imaging instruments for ground photography. From the use of hot air balloons to rockets, a profound understanding of the relationship between the stability of the aircraft attitude and the exposure process has been gained^1^. This reflects the idea of stabilizing the motion characteristics of the carrier to meet the requirements of optical imaging.

Owing to the unmanned nature of powered aerial vehicles, the maneuverability of the aircraft itself has been greatly expanded in the airspace and speed domains, and the imaging system also faces more challenges. In addition to simply stabilizing the line of sight to point to a specific target during the exposure time, the imaging system also needs to isolate the torque interference caused by the aircraft’s flow field, aerodynamic and engine vibrations, and other complex vibrations from multiple sources. Moreover, as the imaging mode changes, it presents different patterns, and the actuation mechanism of the imaging process in forward flight, oblique viewing, and scanning is special compared with that of general optical systems. Therefore, various types of control methods have been studied, and breakthroughs in related methods have greatly improved airborne optical imaging in terms of operational distance and resolution.

In addition to motion factors, drastic changes in internal and external environments can also severely affect image quality. On the one hand, thermal and air pressure environmental changes cause deformation of the optomechanical structure, leading to defocusing of the optical system; on the other hand, the change in the shape of optical elements due to temperature changes cannot be ignored. Compared with defocus, the degradation of image quality caused by shape changes is even more difficult to compensate for. Therefore, in complex and variable environments, reasonable thermal control and structural design must be carried out to ensure that the system can operate normally and obtain high-quality images.

The Changchun Institute of Optics, Fine Mechanics and Physics (CIOMP), Chinese Academy of Sciences, which was founded in 1952, initiated China’s modern optical endeavors and laid a solid foundation for the vigorous development of China’s optical instrument manufacturing industry. In 1958, researchers such as Chen Junren, Wang Runwen, and Zhang Jitang at CIOMP successfully developed a replica of the aerial camera used by the former Soviet Union’s civil aviation, marking the first step in the research of modern airborne optical imaging technology in China. In the 1960s, CIOMP identified 16 key scientific research tasks (the “16 Loong”), including “aerial cameras”, indicating the high level of emphasis CIOMP placed on the field of airborne optical imaging at that time. Concurrently, CIOMP was the first in China to develop a high-altitude camera. In the 1980s, CIOMP developed the DGP series of multispectral aerial cameras, and in the 1990s, it developed China’s first universal small unmanned aerial vehicle (UAV) measurement television camera system, initially meeting the airborne optical imaging needs of UAVs. At the beginning of the 21st century, CIOMP successfully applied digital high-resolution real-time transmission cameras to long-distance high-speed carriers, replacing traditional film-based aerial cameras and completing a technological update. Since the beginning of the 21st century, airborne optics at CIOMP have developed into a relatively independent and comprehensive optical engineering discipline, achieving ultralong-distance oblique imaging. To address dynamic factors, complex working environments, stringent constraints, and increasingly high-performance requirements, CIOMP relies on interdisciplinary integration and integrated innovation, adopting a new approach of “adapting to changes with changes, and remaining unchanged in the face of ever-changing situations” to solve the core challenges of airborne optical imaging.

This article reviews the development trajectory of CIOMP research on airborne optical imaging issues from the dimensions of historical and technological development, with the main content as follows:

1. This study systematically summarizes the difficulties in airborne optical imaging and the innovative contributions of researchers at CIOMP in this field.
2. This study combines the understanding of the multiphysical field mechanisms in airborne optical imaging, clarifies the core scientific questions, and provides key methods and technologies for achieving high-quality dynamic imaging in terms of optical system motion stability, mechanical stability, thermal stability, etc.
3. Future development directions for airborne optical imaging, an interdisciplinary field, are predicted.

In fact, airborne optics has gradually evolved from “seeing” and “seeing clearly” to “distinguishing” and “identifying”. The demand for spectral resolution and polarization information has increased with increasing traction in applications, gradually becoming a direction for future continuous research.

## Results

**Fig. 1:**
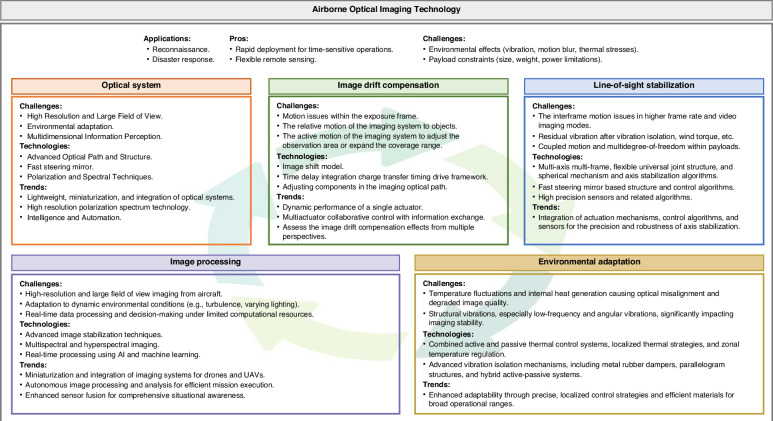
Challenges and key technologies of airborne optical imaging

From simply mounting optical systems on aircraft to systematically establishing the discipline of airborne optics, the mapping relationships among temperature, vibration, motion, and the modulation transfer function (MTF) of optical imaging have been revealed. High-spectral, full-polarization aberration correction methods, active fine anti-interference control methods, “true bit” image shift compensation methods, and wide-temperature adaptability methods have been proposed to address the multiphysical element interference constraints in the imaging process. Many technical breakthroughs have been achieved at the levels of new aiming frame mechanisms, fast steering mirrors (FSMs), and other actuators, as well as from the perspectives of angle and speed measurement and other sensing aspects. A complete set of airborne optical imaging test technologies has been established and developed to simulate extreme conditions, such as low and high temperatures, vibrations, aircraft attitude sway, air pressure changes, long-distance imaging, and target motion. The National Key Laboratory for Dynamic Optical Imaging and Measurement has been established. An imaging technology system has been developed that is adaptable from micro- and small unmanned aerial vehicles to high-altitude high-speed drones, and it has been widely applied in disaster prevention, surveying, and search and rescue. The challenges and key technologies of airborne optical imaging is illustrated in Fig. 1. Since airborne optical imaging is susceptible to environmental effects (vibration, motion blur, thermal stresses, etc), and the payload itself (size, weight, power limitations, etc), the optical system, image drift compensation, line-of-sight stabilization, image processing, and environmental adaptation that associated with high quality imaging are tightly interrelated. These factors must be considered simultaneously when designing an aero-optical payload.

## Discussion

### Airborne optical system imaging techniques

The optical system is the core part of the airborne optical imaging system. Early airborne optical imaging and measurement did not significantly differ from other types of imaging devices, with transmission-type optical systems at the core. By optimizing the structure to reduce higher-order coma, aberration, and lateral chromatic aberration, the requirements for airborne optical imaging and measurement can be met^2,3^.

CIOMP, which is based on common traditional optical schemes, uses a quasi-telecentric imaging optical path to meet the high demands of a large field of view, low distortion, and high-resolution aerial mapping. This method takes into account temperature changes from ground to air environments and image shifts caused by flight. By employing a complex double Gauss quasi-telecentric structure, it achieves a modulation transfer function better than 0.3 across the entire color spectrum within a uniform temperature range of 0–40 °C, with a spatial resolution of 0.08 m@2 km (swath width of 2.4 km)^4^. Compared with the most advanced Leica ADS100 mapping camera of the same period, it increases the coverage width by 43% at the same resolution and is capable of completing 1:500 scale stereoscopic mapping and mapping.

**Fig. 2:**
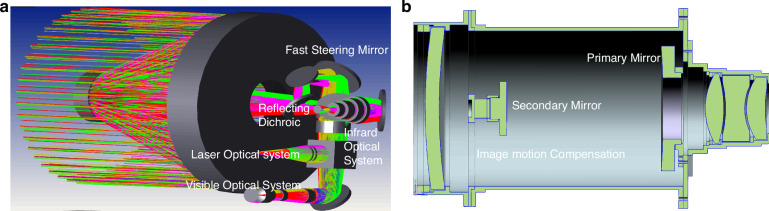
Arrangement of the FSM and its co-aperture optical system^117,118^. **a** FSM device. **b** Configuration diagram of the multiband co-aperture optical system with an FSM

With the continuous enhancement of requirements such as imaging resolution, operational range, field of view, and imaging spectral bands, it is not feasible to achieve effective design using a fixed transmission optical system under the constraints of limited installation space and payload weight in aviation vehicles^5^. CIOMP began to apply reflective optical systems to airborne optical imaging in 2012, incorporating an FSM with controllable deflection angles into a compact anastigmatic optical path, ensuring high-resolution imaging in an airborne motion disturbance environment^6^. In 2023, an innovative aviation catadioptric optical system based on secondary mirror image shift compensation was proposed, achieving a reduction in optical system tolerance sensitivity and realizing an unmanned design, as shown in Fig. 2.

**Fig. 3:**
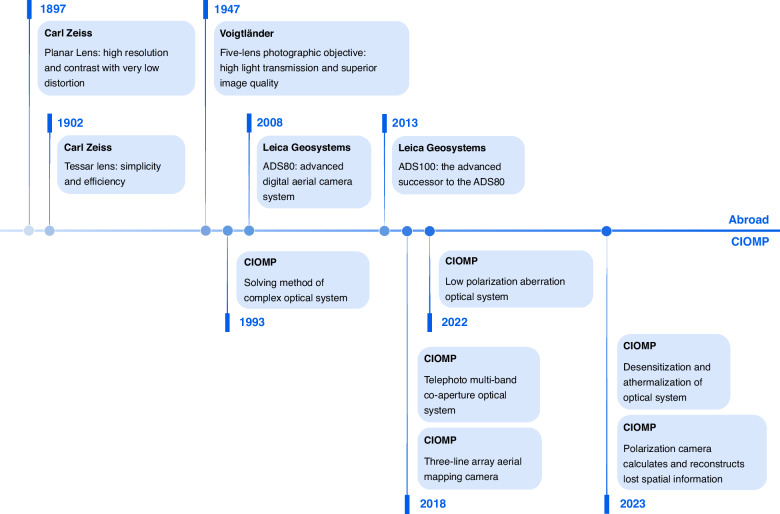
Roadmap of airborne optical system imaging techniques

In recent years, airborne optical imaging has further developed toward the precise perception of multidimensional features to address the diversity of target information, as shown in Fig. 3. The acquired information has expanded beyond traditional intensity information to include spectral and polarization dimensions. Correspondingly, more in-depth research has been conducted in the field of optics. In response to the issue of polarization aberration suppression in optical systems themselves, a method for suppressing polarization aberrations in a catadioptric optical system has been proposed, achieving the design of an optical system with low polarization modulation characteristics^7^; research has been carried out on spectral imaging methods based on polarization modulation filtering schemes, which solve the problem of real-time acquisition of target spectra under air-based dynamic platforms; and the combination of snapshot polarization detection technology and microscanning superresolution technology has realized the accurate recovery of lost multidimensional spatial information^8^.

### Image drift compensation

Airborne optical imaging, which is constrained by dynamic imaging conditions, often experience image shift, which degrades image quality^9^. The reasons for image shift formation are, on the one hand, due to the relative motion of the imaging system to the ground objects during forward flight and, on the other hand, caused by the active motion of the imaging system to adjust the observation area or expand the coverage range. In early imaging systems with short focal lengths and no active optical axis motion, the impact of image shifting was not significant. However, with the improvement of optical system specifications, the relative motion between the optical system and the scene becomes more complex, making its impact on imaging particularly severe.

**Fig. 4:**
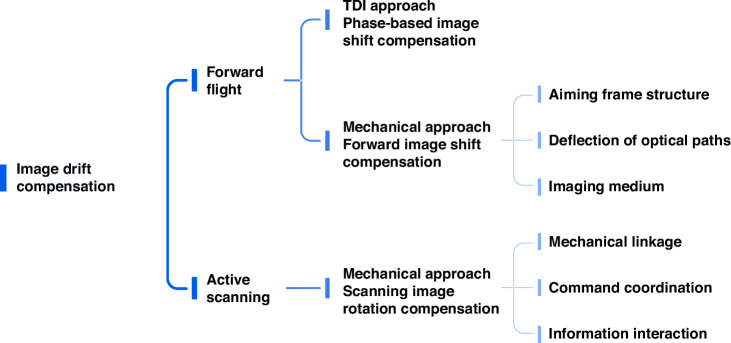
Main development path of image drift compensation

The main development path of image drift compensation is shown in Fig. 4. CIOMP began in-depth research on the characteristics of image shifts in airborne optical imaging in the early 1990s and established an image shift model considering the laws of image formation, representing the forward motion image shift of aerospace imaging systems^10^. Furthermore, to reduce dependence on the geographical coordinates of the photographic target area, Reference^11^ proposed a gray projection method to effectively estimate the image shift velocity, thereby providing an important input reference for image shift compensation.

The implementation of image shift compensation methods involves various means to match the exposure process of the imaging photosensitive medium (CCD, CMOS, film, etc.) with the image shift speed. Especially with the development of digital devices, the time delay integration (TDI) function provides excellent support for image shift compensation. By controlling the interline charge transfer speed of the CCD and CMOS cameras in the TDI working mode to match the image shift speed, the image shift can be compensated^12^. However, the imaging efficiency of this method is limited by the charge readout speed and imaging frame rate, and the compensation performance is affected by factors such as control system precision, bandwidth, and sensor resolution, making it difficult to achieve a complete match between the interline charge speed and the scene image point speed. In response to the aforementioned issues, CIOMP has proposed a new TDI CCD charge transfer timing drive framework, revealing the “true bit” image shift compensation principle, which solves the impact of inherent image shift on imaging quality caused by TDI CCD charge transfer, making the inherent image shift caused by charge transfer better than the 1/2Ф pixel size^13^.

For push-broom-type electro-optical imaging payloads, the accuracy of forward image shift compensation can be improved by precisely acquiring aircraft speed‒height ratio information. Based on the method of coordinate transformation, ref. ^14^ analyzed the image shift compensation rule based on the FSM and designed the corresponding forward image shift compensation scheme. For a scanning-type electro-optical imaging payload, the image shift caused by active movement can be compensated for by precisely matching the scanning mirror and camera speeds^15^. The mechanical method of compensation has obvious advantages in adapting to area array imaging and improving the matching accuracy of image shift compensation. CIOMP has proposed methods for compensating for image shift caused by continuous scanning of step-state systems using FSMs and for compensating for image shift by driving the motion of the imaging focal plane components with piezoelectric ceramic actuators, which can achieve an image shift compensation accuracy far less than 1/3 the pixel size^16,17^.

Additionally, the imaging process of panning and scanning to expand the field of view not only results in an image shift, but also introduces significant image rotation, when a scanning reflective mirror with an acute angle between the mirror normal and the optical axis is employed. This rotation also requires compensation. Reference^18^ used spatial geometric optics to establish a model for image rotation in aerial cameras and gradually developed an electric control de-rotation scheme based on a dual-motor drive and independent control circuits, which meets the precision imaging requirements of a certain airborne optical remote sensor. The L2 norm of the synchronization error between components was reduced by 99.83%^19^.

For future image shift compensation control, on the one hand, it is necessary to start with a single actuator, enhancing the actuator’s dynamic performance and anti-interference capabilities under high-dynamic environments and complex nonlinear constraints. On the other hand, it is also essential to consider multiactuator collaborative control, leveraging the topological structure of information exchange among multiple actuators and collaborative control strategies to further improve the performance of image shift and image rotation compensation. Additionally, the compensation effects should be comprehensively assessed from multiple perspectives, including control and imaging.

### Line-of-sight stabilization control

Compared with image shift compensation, which addresses motion issues within the exposure frame, line-of-sight stabilization primarily addresses interframe motion issues in higher frame rate and video imaging modes^20^. The most common method is to mount an optical system on a mechanical gimbal, achieving line-of-sight stabilization through control of the gimbal. The stability precision of the imaging system greatly affects the photography distance. Early gimbals used two rotational axes to adjust the azimuth and pitch attitudes. Since the platform was directly exposed to the external environment, during the flight of the carrier aircraft, the wind resistance would generate significant disturbing torques, leading to a stabilization precision of the two-axis gimbal that could only reach the milli-radian level.

**Fig. 5:**
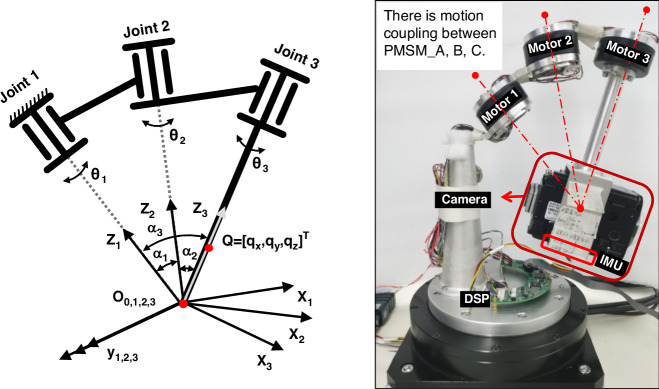
Schematic diagram of the inner frame structure of a spherical mechanism-based stabilization platform^24^

**Fig. 6:**
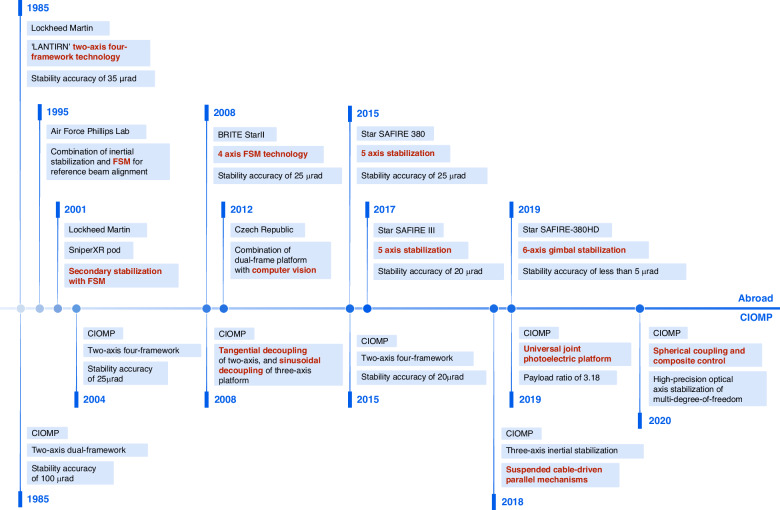
A typical development roadmap of line-of-sight stabilization control for airborne electro-optical payloads

CIOMP proposed a two-axis four-frame structure in the 1990s, with a stability precision of 15-25 μrad^21^. The outer frame serves to extend the rotation range and isolate external disturbances; the inner frame independently performs high-precision attitude control. However, the two-axis gimbal cannot effectively compensate for the attitude changes in the roll direction of the carrier aircraft. In recent years, new flexible universal joint structures and spherical mechanisms have been developed to construct a three-degree-of-freedom inner frame, which, combined with a two-axis outer frame, can solve this problem^22^. To this end, CIOMP established a kinematic model of a three-axis spherical gimbal mechanism and conducted experimental verification^23^. Furthermore, a sliding mode-assisted active disturbance rejection control method was proposed to address the control issues of coupled motion between axes^24^. This enables multidegree-of-freedom, high-precision line-of-sight stabilization within a limited space, as illustrated in Fig. 5. The typical development roadmap of line-of-sight stabilization control for airborne electro-optical payloads is shown in Fig. 6.

**Fig. 7:**
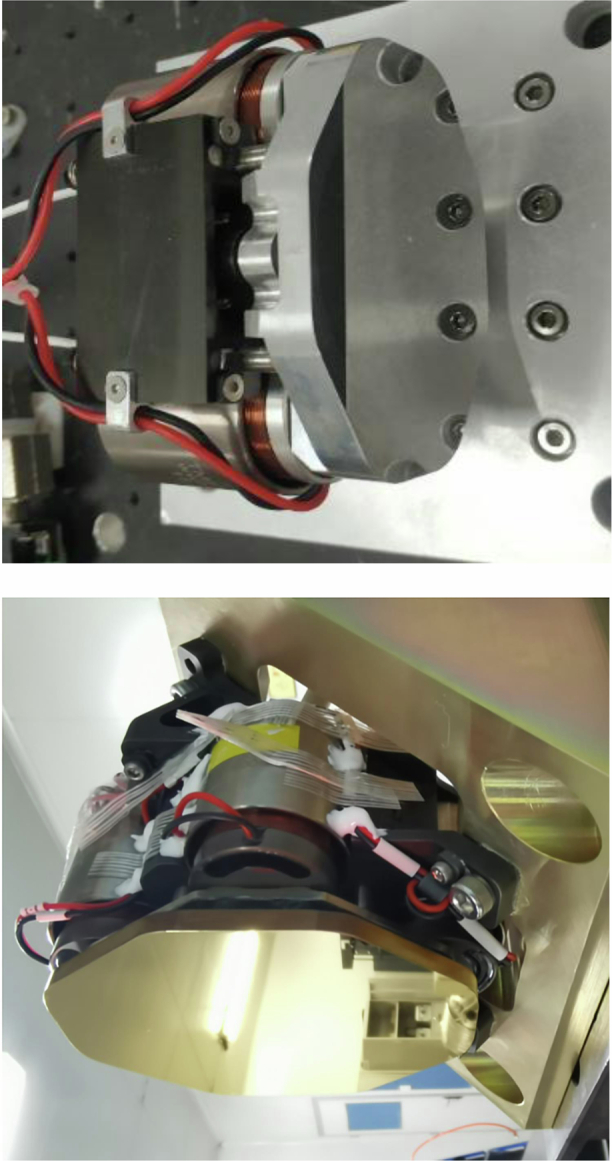
Typical FSMs developed via CIOMP^29^

On the one hand, line-of-sight stabilization control, which is independent of the development of gimbals, is based on the deployment of FSMs. CIOMP research on FSMs began with high-precision tracking and measurement requirements for acceleration targets on large-aperture ground-based telescopes^25^ and gradually expanded to include mobile platforms such as vehicle-mounted and airborne systems^26,27^. In terms of the kinematics of the FSMs themselves, both rigid support^28^ and flexible bearing structures for FSMs have been explored^29^. Leveraging the frictionless motion axes and wide response bandwidth characteristics of the FSMs themselves, CIOMP has enhanced the line-of-sight stabilization precision of airborne optical imaging systems to the 5 μrad level. Typical FSMs developed by CIOMP are shown in Fig. 7.

On the other hand, the kinematic resolution of mechanical frameworks requires the use of shaft angle sensors to measure the relative rotation angles between frames, making encoders a crucial component affecting the high-precision line-of-sight pointing control of imaging payloads. CIOMP developed a 23-bit optoelectronic shaft angle encoder with a resolution of 0.15 arcseconds and an angular measurement accuracy mean square value better than 0.6 arcseconds as early as 1985^30^, which was successfully applied in various types of electro-optical payloads. However, the limitations of optoelectronic encoders in terms of environmental adaptability, service life, power consumption, and cost have restricted their application. Considering these factors, CIOMP has developed new types of encoders, such as magnetic encoders with strong anti-interference capabilities and adaptability to harsh environments^31^, and interferometric encoders with high-precision and high-resolution^32^, each of which play different roles in the control of various electro-optical imaging payloads.

**Fig. 8:**
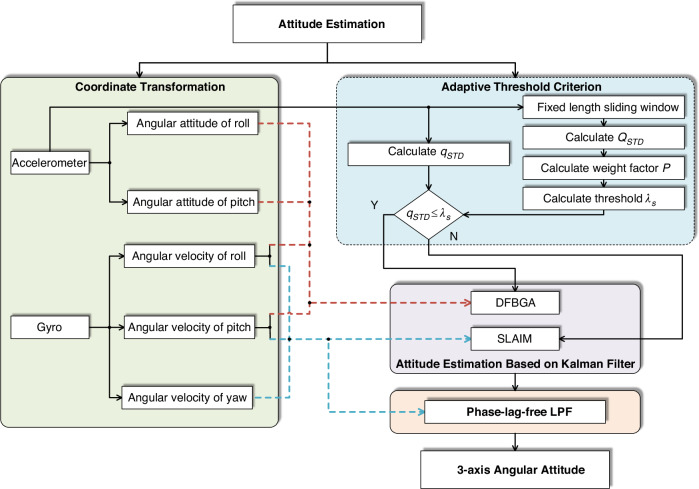
Block diagram of the low-phase lag multi-inertial sensor fusion algorithm^33^

Optical encoders are used to measure the relative rotation angles of the gimbal. But to achieve line-of-sight stabilization control, it is also necessary to measure the motion in the inertial space to construct feedback. Gyroscopes can obtain the angular velocity of the imaging payload relative to the inertial space and are commonly used to construct line-of-sight stabilization loops. With the increasing demands for the imaging performance of optical payloads, control systems that rely solely on information from a single sensor are gradually unable to meet the system’s tracking precision and disturbance rejection requirements. Multiple closed-loop control systems that utilize data from multiple sensors have emerged. Fusing data from different types of sensors can effectively broaden the data bandwidth and improve the data quality. Reference^33^ designed a data fusion algorithm based on gyroscopes and accelerometers and applied a phase lag-free low-pass filter to compensate for the phase lag errors introduced, reducing the root mean square error of the inertial angular velocity signal by more than 44%. The algorithm framework is shown in Fig. 8.

In addition to directly measuring motion information using sensors, it is also necessary to obtain the differential signals of the sensors to construct various advanced control algorithms. To address the issue that direct differentiation methods amplify high-frequency noise of the signal, leading to a decline in signal quality, a nonlinear tracking differentiator with feedforward, as shown in Eq. (1)^34^, has been proposed. By introducing feedforward of the input signal when solving the differential equation, this method significantly improves the accuracy of the differential estimation and enhances the quality of the differential signal.

1 $$\left\{\begin{array}{l}{\dot{x}}_{1R}(t)={x}_{2R}(t),{x}_{1R}(0)={x}_{10},{x}_{2R}(0)={x}_{20}\\{\dot{x}}_{2R}(t)=-{R}_{\alpha 1}^{2} \{ {\beta }^{\frac{p}{q}}{[{x}_{1R}(t)-r(t)]}^{\frac{p}{q}}+\left[\right.{x}_{1R}(t)-r(t)\}-{R}_{\alpha 2}^{2}\left\{{\beta }^{\frac{p}{q}}{[\frac{{x}_{2R}(t)}{R}]}^{\frac{p}{q}}\right.\\\quad\quad\quad\quad\left.+\frac{{x}_{2R}(t)}{R}\right\}+\alpha \dot{r}(t)\end{array}\right.$$

In summary, the control of line-of-sight stabilization relies on actuation mechanisms, control algorithms, and sensors. However, advanced control algorithms have not been widely used in engineering. The design of systematic and high-performance optical line-of-sight stabilization control methods will also be one of the key technologies that CIOMP will focus on in the future. In addition, as the requirements for imaging distance increase, methods to increase performance in all three aspects are needed. And through integrated application, the precision and robustness of line-of-sight stabilization in airborne optical systems continuously improve.

### Image processing methods for aerial imagery

**Fig. 9:**
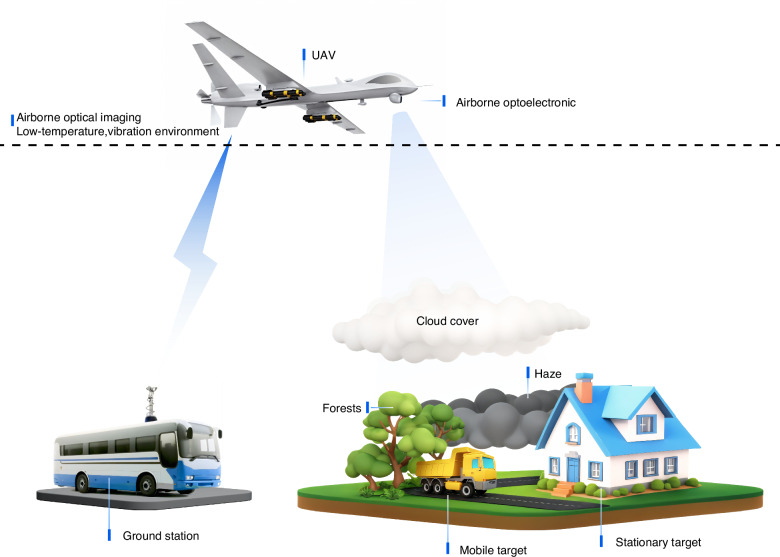
Actual application scenarios of airborne electro-optical payload target tracking^35^

Digitalization, as one of the hallmark advancements in modern airborne optical imaging technology, has led to the flourishing study of computer processing methods for airborne optical imaging. The focus of early image processing was on achieving automatic detection and tracking of imaging targets. The algorithms were used to calculate in real time the number of pixels by which the target deviates from the center of the image (off-target amount), and the data were sent to the servo system to form a closed-loop stable tracking system^35^, as shown in Fig. 9. After the 1980s, systems such as the Model 975 electro-optical tracking system, the the “Sea Dart II” type, the TOTEM and Volcan types developed by the French CSEE company and SAGEM company, the MSIS system developed by the Israeli Elop company, and the “Sea Guardian” system developed by the Swiss Contraves company integrated several types of sensors onto the same turntable and achieved real-time target tracking^36^.

Unlike conventional target tracking algorithms, electro-optical pods require extremely high real-time performance to achieve high stability accuracy. As early as the 1990s, CIOMP introduced a servo stabilization platform based on image tracking and achieved real-time tracking. Initially, tracking algorithms were based mainly on correlation tracking and centroid tracking. The MOSSE algorithm^37^ and KCF algorithm^38^ quickly became standard methods in the field of target tracking, effectively achieving a good balance between speed and accuracy. The KCF algorithm subsequently further improved its performance by introducing gradient histogram features and scale adaptive processing^39^.

**Fig. 10:**
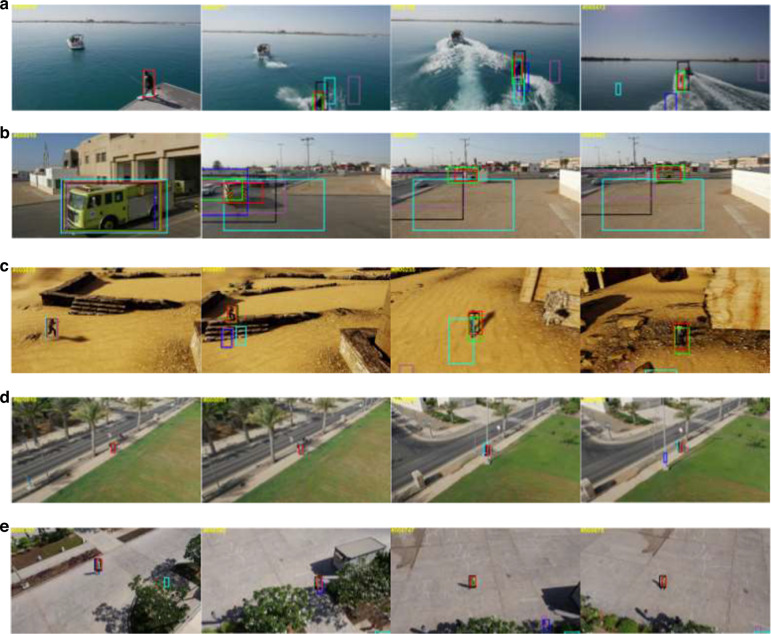
Single-object tracking effect demonstration^43^. (**a**) Waterskiing, (**b**) truck, (**c**) person, (**d**) crowd, (**e**) person

**Fig. 11:**
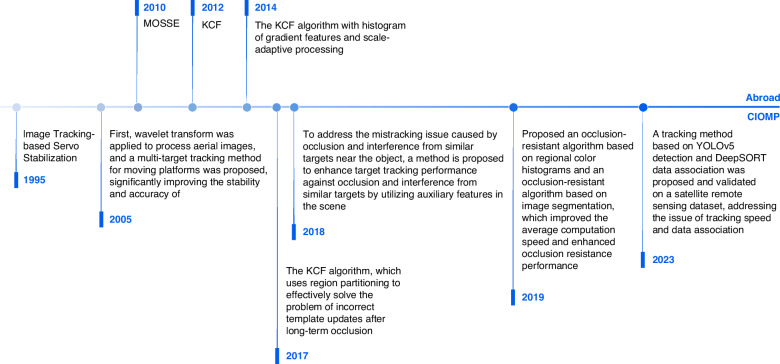
The development history of image tracking

CIOMP also rapidly implemented the engineering of related algorithms and achieved unique breakthroughs in special scenarios of electro-optical payloads, such as target occlusion, tracking accuracy, and low signal-to-noise ratio (SNR) issues, especially showing unique innovative advantages in high-precision and high-stability target tracking technology. To address tracking errors caused by complex target motion, dynamic observational features, and challenging backgrounds, a particle filter tracking method has been proposed. This method integrates an acceleration-based two-step dynamic model, an asymmetric kernel function, image segmentation algorithms, and a random forest classifier. The experimental results demonstrate that this approach effectively enhances tracking accuracy and resolves tracking issues in complex background scenarios^40^. To solve the problem of target loss and recapture caused by short-term target occlusion, the conventional KCF algorithm was modified to adopt a regional division approach, effectively solving the problem of the template being incorrectly updated after long-term occlusion^41^. To address issues such as long shooting distance, low target resolution, and a low signal-to-noise ratio, the wavelet transform was first used to process aerial images, and a moving platform multitarget tracking method was proposed, which significantly enhances the stability and accuracy of target tracking^42^. In recent years, CIOMP has continuously advanced the innovation of tracking algorithms. For example, in 2023, a tracking method based on YOLOv5 detection and DeepSORT data association was proposed and verified on aerospace remote sensing datasets, solving the problems of tracking speed and data association^43^, as shown in Fig. 10. These studies further promote the application research and technological innovation of CIOMP in the field of target tracking. The development history of image tracking is shown in Fig. 11.

In recent years, researchers from both domestic and international communities have advanced the technology for denoising and defogging aerial images under hazy conditions, resulting in a clear developmental trajectory. Internationally, in 2010, an adaptive algorithm based on the regional similar transmission assumption effectively achieved image denoising^44^; in terms of improving computational efficiency, a method combining the dark channel prior and Gaussian filter rapidly and effectively achieved defogging of remote sensing images^45^; in 2016, an algorithm based on the dark channel prior was optimized through edge extension and region-guided filtering^46^, which also significantly reduced computational time; in 2020, by utilizing densely connected pyramid networks and U-net networks^47^, the transmission map and atmospheric light values were jointly optimized, effectively enhancing image details and realism; and in 2022, the DRL_Dehaze network based on multiagent deep reinforcement learning^48^ achieved precise defogging for different ground types and fog levels. In the same year, a double-scale transmission optimization strategy combined with a haze-line prior algorithm addressed issues such as loss of texture details and color distortion^49^. In 2024, a lightweight defogging framework was proposed^50^, which combines density awareness, cross-scale collaboration, and feature fusion modules to efficiently process aerial images with varying fog densities.

**Fig. 12:**
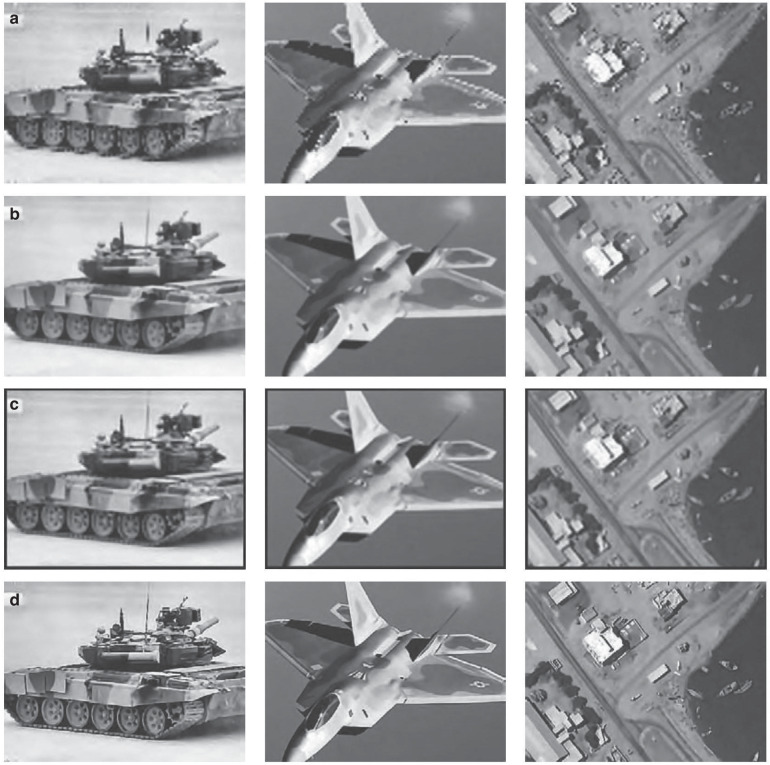
Efficacy of the superresolution algorithm for aerial images based on multiphase group reconstruction^56^. **a** Bicubic results. **b** Results of MAP + L2 + BTV. **c** Results of ScSR. **d** Results of the proposed method

CIOMP has also achieved a series of innovative results in terms of haze removal and noise reduction in aerial images. In 2012, Reference^51^ conducted research from the perspectives of physical haze removal and image haze removal and proposed a method for rapid haze removal and clarity recovery. In 2013, methods for blind restoration and evaluation of unknown or partially known blurred images were proposed. These include defocus blur radius estimation based on the Hough transform, a supervariational regularization blind restoration algorithm, and a new no-reference quality assessment method. The experimental results show that the proposed methods perform well in terms of restoration accuracy, stability, and visual effects^52^. In 2013, Reference^53^ proposed a haze removal method for oblique remote sensing images in high-altitude and long-distance aerial camera imaging, reducing the processing time to 15% of the original value and significantly improving the image clarity evaluation indicators. In addition, in the field of superresolution reconstruction, in 2015, Reference^54^ analyzed the significance of superresolution technology in improving the imaging quality of aerial images; in 2016, References^55,56^ conducted in-depth research on the key technologies of superresolution reconstruction of aerial images and successfully achieved engineering application, as shown in Fig. 12.

**Fig. 13:**
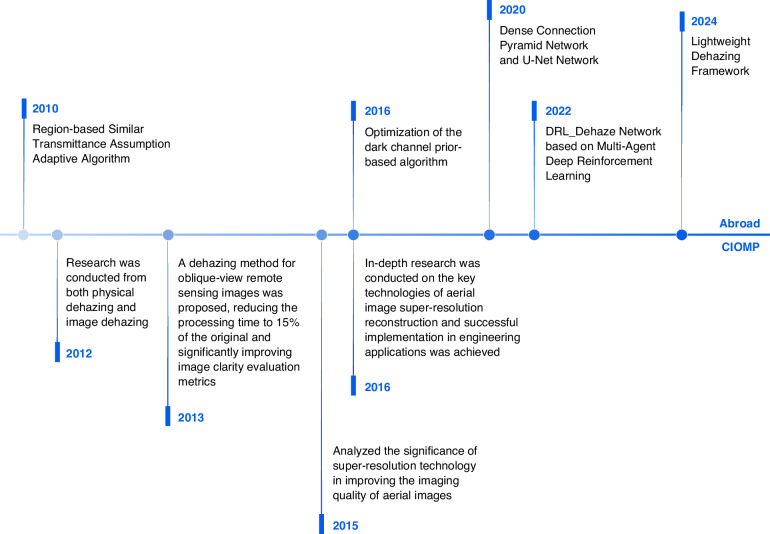
Development journey of image sharpening

Overall, from the theoretical innovation of international research to engineering optimization within CIOMP, which combines actual needs, the development in this field has gradually shown a complete route from theoretical models to engineering applications. By comparison, the research at CIOMP has advantages in rapid processing and detail optimization, providing important support for the practical application of haze removal and superresolution reconstruction technology in aerial images and promoting the development of this field toward high efficiency and precision. The development journey of image sharpening is shown in Fig. 13.

Image fusion is one of the current research hotspots, and the complementarity and redundancy between various optoelectronic sensors can address the issue of incomplete or inaccurate information from a single imaging sensor. The fused output image effectively leverages the advantages of different spectral bands, facilitating operators in better detecting and identifying targets. Additionally, utilizing the complementarity of multiple pieces of information to expand the system’s perception of spatiotemporal coverage is also an important means for military reconnaissance, recognizing camouflage, and achieving optoelectronic countermeasures.

Starting in 2006, the image fusion model based on OIF and wavelet transform for hyperspectral data and aerial imagery marked the beginning of image fusion technology^57^. In 2007, a land cover classification method based on an SVM that fuses high-resolution aerial imagery and LIDAR data improved classification accuracy^58^. In 2009, the fusion algorithm of LiDAR and aerial imagery effectively increased the accuracy of building footprint extraction^59^. In terms of improving the fusion effects, the low computational cost image fusion algorithm proposed in 2010^60^, the two-scale image fusion method using guided filtering in 2014^61^, and the deep convolutional neural network combined with PAN and MS images in 2018^62^ provided higher-resolution fused images.

**Fig. 14:**
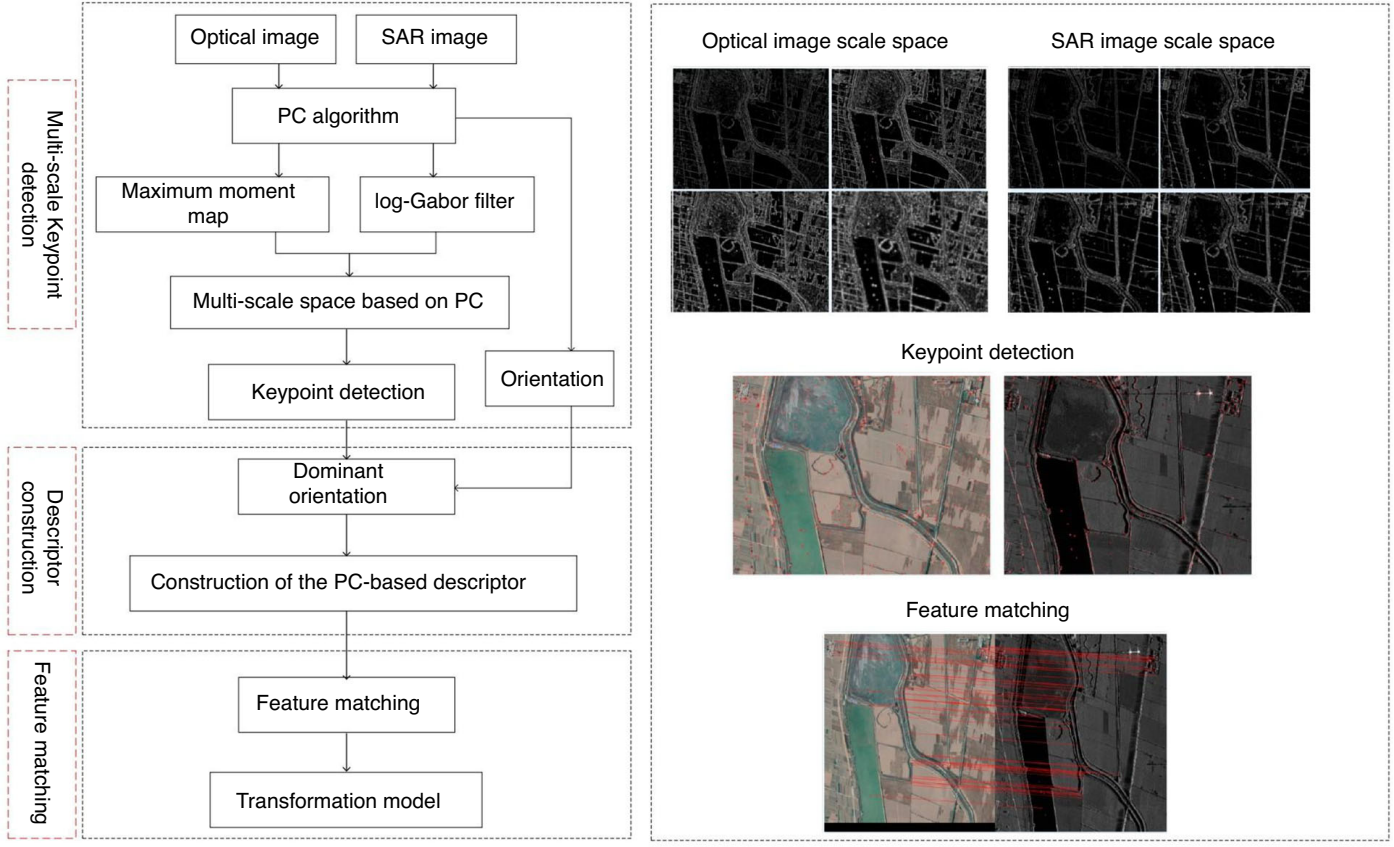
Algorithm flowchart proposed by Xie Zhihua^70^

Moreover, CIOMP has conducted extensive research in the field of image fusion, promoting the innovation and application of image fusion technology^63–66^. In 2010, to address the motion blur problem in aerial reconnaissance, several image restoration algorithms, including the one-dimensional Wiener filter (1DWF) algorithm for simplifying computations, methods for recovering oblique and rotational motion blur, and multibur image restoration techniques, were proposed. The fast Fourier transform (FFT) algorithm was improved to increase computational efficiency, and GPU technology was employed to accelerate the image restoration process. This led to real-time restoration, significantly improving both processing speed and efficiency^67^. In 2012, a method was proposed to address real-time and accuracy issues in visible and infrared image registration and fusion. The method utilized an optimized particle swarm algorithm for registration, combined with multiscale image fusion and wavelet transform techniques. The results included improvements in registration accuracy, image fusion quality, and real-time performance, effectively solving the problems of high-frame-rate reconnaissance image registration and fusion^68^. From 2017 to 2023, in-depth research was conducted on the registration algorithms and efficiency of airborne visible and infrared images, achieving fast registration and fusion of visible and infrared images^63–66^. With the continuous development of airborne payload types, image fusion technology has gradually evolved from early optical image fusion to the fusion of optical and SAR radar images. CIOMP has also made breakthroughs in this field. From 2021 to 2022, References^69,70^ studied the registration technology of visible light and SAR remote sensing images and implemented and verified these technologies on embedded platforms, promoting the practical application of image fusion technology, as shown in Fig. 14.

**Fig. 15:**
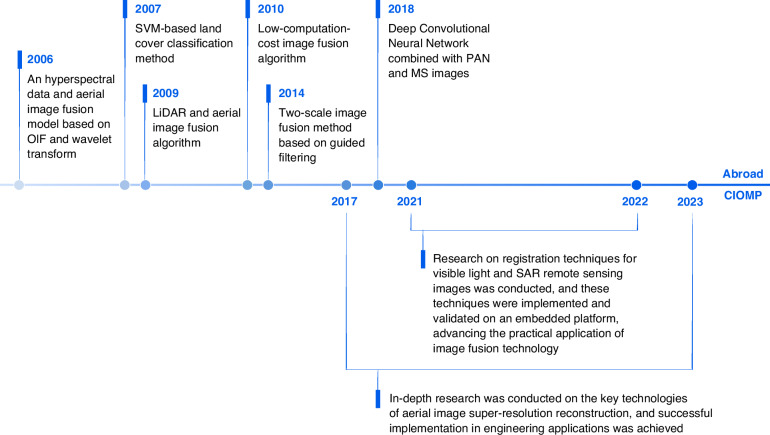
Evolutionary journey of image fusion development

Through this journey, we can observe that image fusion technology has evolved from its initial simple image synthesis to the current integration of multisensor, multimodal data, traversing multiple technological milestones. Compared with foreign standards, the innovation of CIOMP is characterized by its close alignment with specific application requirements, progressively achieving the fusion of optical images with multimodal images (including SAR and visible light images). This not only enhances the resolution and recognition accuracy of the images but also propels the efficient application of image fusion technology, particularly in areas such as military reconnaissance and remote sensing monitoring, where significant practical achievements have been made. The evolutionary journey of image fusion development is shown in Fig. 15.

In recent years, target recognition technology has become a hot topic in the field of aerial optoelectronic image processing and has undergone rapid evolution from traditional detection algorithms to deep learning methods. Traditional detection algorithms rely mainly on manually designed features. For example, the Viola‒Jones (VJ) detection algorithm^71^ uses Haar features and an AdaBoost cascade classifier to achieve target detection, but it lacks adaptability to complex backgrounds. In 2008, the deformable part model (DPM) algorithm based on histogram of oriented gradients (HOG) features was proposed^72,73^, which improves detection efficiency through part-based modeling, but it is still limited by the generalizability of manually designed features.

With the rise of deep learning technology, target recognition has entered a new phase. The R-CNN series (R-CNN^74^, Fast R-CNN^75^, Faster R-CNN^76^, and Cascade R-CNN^77^) achieves high-precision target detection through a two-stage detection strategy but with high computational complexity. To address the speed issue, single-stage detection networks such as RetinaNet^78^, RefineDet^79^, and the YOLO series^80^ emerged, significantly improving the detection speed through direct classification and bounding box regression.

In recent years, researchers have proposed innovative methods that target the characteristics of weak and small target detection. For example, Stitcher expands small target samples through data augmentation^81^, SOD-MTGAN leverages GAN applications in the superresolution field to enhance target resolution^82^, ION enhances environmental perception through multiscale contextual information^83^, and attention-based dual-path modules further highlight key features and suppress background noise^84^.

Compared with international advanced levels, CIOMP has demonstrated significant innovation and engineering application capabilities in the field of target recognition technology. In terms of traditional algorithm research, in response to issues such as scale, perspective, and lighting changes in remote sensing images, a robust and fast feature point detection algorithm based on scale space has been proposed^85^. From 2018 to 2020, traditional algorithms^86–88^ were used to identify targets such as aircraft and ships. In the era of deep learning, CIOMP closely follows technological frontiers and, in combination with embedded low-power hardware platforms, has led to the development of various practical algorithms. From 2018 to 2020, algorithms^89,90^ based on deep learning and automatic identification of ship targets in embedded systems were developed and experimentally verified. In 2023, Reference^91^ utilized the spatial and spectral characteristics of hyperspectral images, and methods for target detection in complex aerial environments were proposed and applied in engineering projects. In 2023, rapid detection of aerial small targets and actual deployment using improved YOLOv5 were achieved^92^.

Compared with the international level, CIOMP has unique advantages in optimizing embedded deep learning algorithms, combining hyperspectral imaging with target recognition, and detecting weak and small targets in complex environments. The related technologies have been successfully applied to multiple types of airborne optoelectronic payloads, providing significant support for both the defense and civilian fields.

**Fig. 16:**
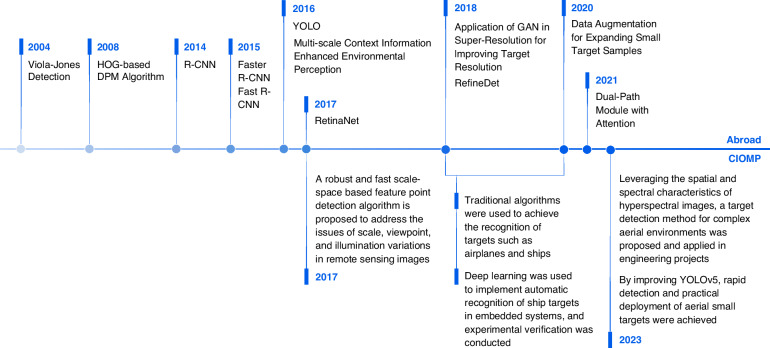
Development history of object detection

With the development of optoelectronic payloads toward all-weather, multispectral, and multimode detection systems, the image processing technology of optoelectronic devices has evolved from a singular focus on target capture and tracking functions to intelligent, multimodal fusion and quantitative analysis. This evolution has resulted in clearer output images, more convenient target capture and tracking, and more precise target localization^93^. The development history of object detection is shown in Fig. 16.

### Environmental adaptation methods

#### Thermal adaptation methods

**Fig. 17:**
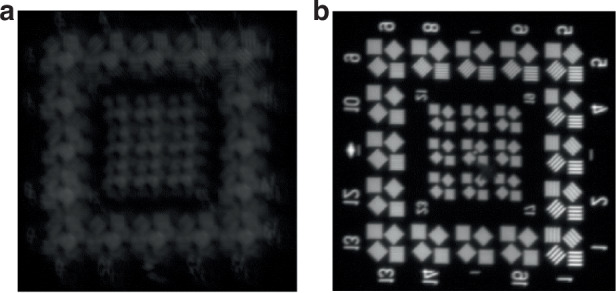
Imaging of the target under different conditions^94^. **a** Without active control. **b** With active control

During the operation of airborne optical imaging systems, drastic changes in external environmental temperatures and heat generated by internal equipment can cause errors in the structure and optical system, significantly impacting image quality. CIOMP conducted an experimental evaluation of the imaging resolution comparison between scenarios with and without thermal control^94^, clearly demonstrating that in complex and variable environments, a reasonable thermal control design must be implemented to ensure that the system operates normally and captures high-quality images, as shown in Fig. 17.

The thermal control design in aviation often draws on the environmental control approach used in space cameras^95–97^, which employs both active and passive measures to regulate temperature and ensure that the optical system remains stable in the aviation environment, similar to ground conditions, thus allowing the camera to achieve high-resolution imaging. In 1980, the thermal control systems of the U.S. KS146 and KS-127A aviation electro-optical platforms used a combination of active and passive thermal control methods^98,99^. During normal flight operations, the KS146 thermal control system could maintain the overall temperature of the optical lens within a range of ±1.1 °C, thereby eliminating the impact of temperature fluctuations on image quality and enhancing imaging resolution.

Similarly, in the early airborne imaging systems of CIOMP, a control strategy was employed that primarily relied on passive control with active control as a supplementary measure^96,97,100^. By establishing a finite element thermal model of the camera and performing calculations based on aerospace environmental boundary conditions, the temperature distribution of the model was obtained, allowing targeted thermal insulation measures to be taken. Experiments have shown that under conditions ranging from -45 °C to 20 °C, both the axial temperature difference and the radial temperature difference for which the focusing mechanism has difficulty compensating are less than 5 °C. A thermal control strategy that balances low power consumption with high MTF has been proposed, addressing the conflict between these two requirements^101^.

**Fig. 18:**
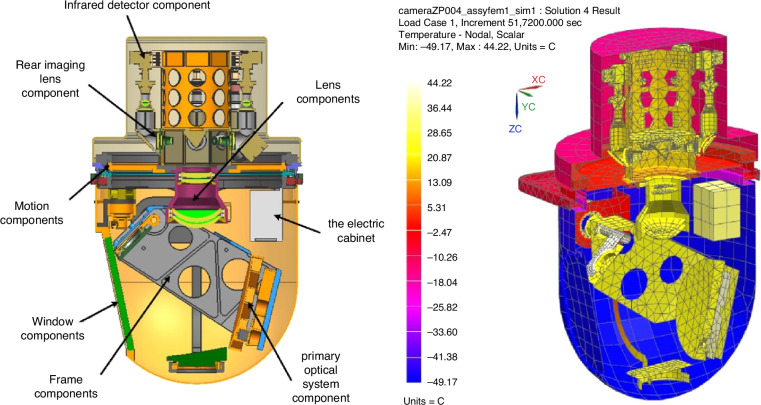
Schematic diagram of the aerial camera structure and regional thermal control temperature field^102^

However, as the focal length of optical systems increases and their size increases, conducting comprehensive thermal control for the entire imaging system presents issues such as low precision and high power consumption. CIOMP has gradually developed into a localized thermal control method. This method involves the use of different thermal control strategies for parts of the optical system, lens groups, and detectors, which have significantly different thermal effects and thermal sensitivities. Additionally, the connection between the detector and the frame is achieved using phase change materials (octadecane) to facilitate heat conduction, whereas active thermal control measures, such as polyimide heating sheets, are used for temperature compensation. As a result, in an environment ranging from -40 °C to 50 °C, after 2 hours of operation, the temperature range of the optical system is 18.5 °C to 22.2 °C, the temperature range of the lens group is 19.1 °C to 20.3 °C, and the temperature range of the detector module is 19.7 °C to 31.9 °C. This zonal thermal control strategy has been applied in multiple airborne imaging systems^102^, as shown in Fig. 18.

**Fig. 19:**
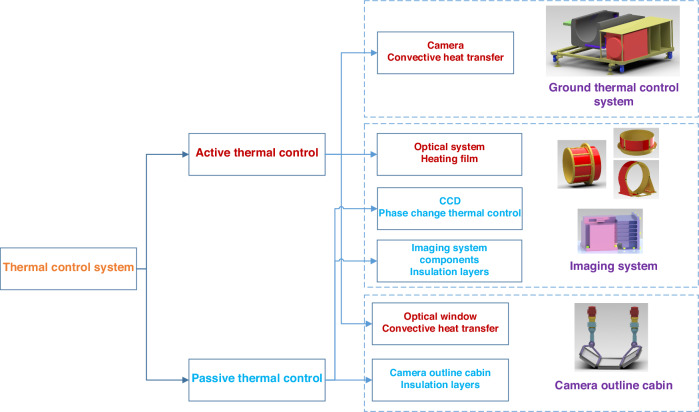
Schematic diagram of ground thermal control and camera system-level thermal control^103^

Recently, CIOMP has proposed a multilayer system-level thermal control method in which an airborne camera is divided into an imaging system and a contour cabin. These two parts are connected through materials with low thermal conductivity, creating an air insulating layer in between. The total power consumption of the thermal control system is 270 W. High-quality images can be obtained when the optical lens temperature gradient is less than 5 °C and the CCD temperature is below 30 °C. Compared with traditional thermal control methods, this approach effectively reduces power consumption and simplifies the difficulty of thermal control^103^, as shown in Fig. 19.

#### Vibrations

The structural vibration of optoelectronic device carriers involves vibrations of different magnitudes at frequencies ranging from 5 to 2000 Hz. In fact, when the focal length of the optical system is 0.3 m and the object distance is 3000 m, the image shift caused by angular vibration with an amplitude of 30” is 436 times that caused by linear vibration with an amplitude of 1 mm. The impact of angular vibration on the imaging quality of optoelectronic pods is far greater than that of linear vibration^104^. Therefore, various active and passive vibration isolation methods that target the suppression of angular vibration in aerial cameras, including different vibration isolation modes, principles, materials, and structural forms, have been proposed.

CIOMP initially extensively explored the materials used for vibration dampers in aerial cameras. Two types of small metal rubber dampers, which utilize metal rubber as the vibration isolation element, were developed to limit certain degrees of freedom. These dampers were embedded in the vibration reduction systems of both the inner and outer frames of the optoelectronic pod, designing a two-stage vibration reduction system that theoretically achieves angular displacement isolation in three directions^105^. Furthermore, considering the coupling and distribution issues among multiple isolators, microangular displacement isolation technology based on parallelogram and three-directional equal stiffness mechanisms was proposed by applying a damping-variable three-directional equal stiffness isolator within a parallelogram mechanism equipped with X, Y, and Z directional guide rails. The angular displacement generated during vibration is converted into linear displacement through the parallelogram mechanism^106^.

**Fig. 20:**
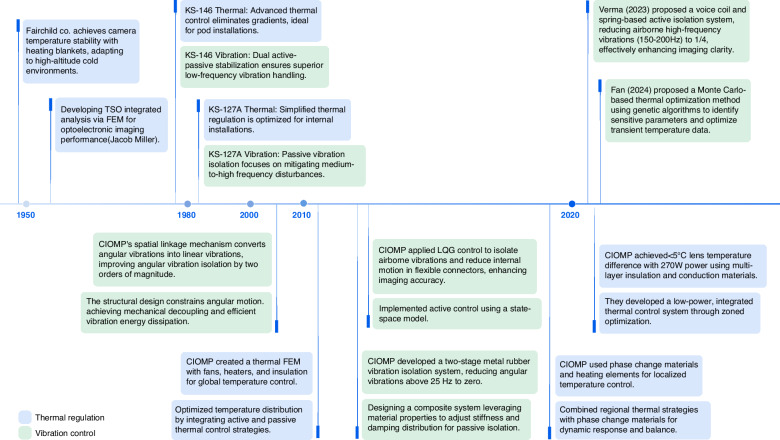
Roadmap of the environmental adaptation methods

Passive damping methods based on mechanical vibration isolation structures can effectively filter high-frequency vibrations and suppress angular vibrations, but it is difficult to isolate the impact of low-frequency vibrations below 10 Hz on the payload. Active vibration isolation methods can provide better compensation for low-frequency vibrations, ensuring the operational performance of optical payloads over a wider frequency range of vibrations^107^. However, the added active vibration control systems also increase the load on the aircraft and therefore are less commonly applied to airborne optoelectronic equipment at present. In the future, the development of compact, low-power, and high-efficiency active‒passive composite vibration isolation remains a challenging issue that must be addressed in airborne optical imaging. A roadmap of environmental adaptation methods is shown in Fig. 20.

### Experimental verifications

**Fig. 21:**
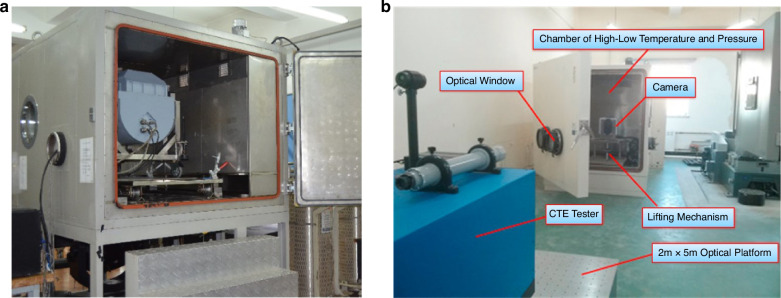
Thermal-Optical Experimental Setup for Imaging Resolution. **a** Imaging resolution measurement under controllable temperature and pressure through a steering mirror^103^. **b** Conducting thermal‒optical experimental verification through the expansion of temperature and pressure test chambers^101^

Airborne optical imaging operates in a unique environment, and to ensure the flawless execution of every flight imaging mission, it is necessary to establish a comprehensive ground testing environment. Currently, China has formulated multiple ground testing standards^108,109^. The design of environmentally adaptable testing equipment for airborne optical equipment is often based on testing standards or specific airborne environments. CIOMP has established a measurement system that not only meets various national and industry standards to ensure standardized product environmental adaptability testing but can also complete a variety of specific flight profile experimental validations^101,103^, as shown in Fig. 21.

The simulation of motion and vibration is a crucial step in ensuring the reliable performance of equipment during flight. Vibration tables generate controllable linear or multiaxis vibrations through electromagnetic shakers or hydraulic systems. In the testing of aerial cameras, these devices simulate vibrations in longitudinal, lateral, and vertical directions to assess the equipment’s response at specific vibration frequencies. The attitude of the aerial payload changes with the adjustment of the carrier aircraft, exhibiting strong dynamics. In fact, the attitude of the carrier aircraft can affect the vibration vectors. To address this, an optical imaging function module has been expanded on the basis of the vibration table using a parallel light pipe, taking into account the impact of the carrier platform’s vibration on the optical imaging MTF. The resulting special environment simulation optical experimental verification device can meet the imaging and measurement needs of airborne optical systems^110^.

### Applications

In 1958, CIOMP began to undertake the development task of reconnaissance cameras for MiG aircraft, marking the first step in the development of China’s aerial reconnaissance technology. In the 1980s, CIOMP developed the DGP series of multispectral aerial cameras. In the 1990s, the first general-purpose small unmanned aerial vehicle (UAV) measurement television imaging system was developed in China to meet the airborne optical imaging needs of UAVs. At the beginning of the 21st century, CIOMP successfully applied digital high-resolution real-time reconnaissance equipment to long-distance high-speed carriers, replacing traditional film-based aerial reconnaissance cameras and completing technological updates and upgrades.

Since the beginning of the 21st century, with the development of drone technology, aerial remote sensing technology has been increasingly integrated into various fields, such as geological surveys, disaster management, and agriculture. The first large-scale application of aerial photography for geological surveys in China was during the 2008 Wenchuan earthquake, where preliminary geological survey results of the affected areas were rapidly obtained^49^.

In the field of geological surveys, CIOMP was commissioned by the Beijing Geographic Institute in the 1970s to develop a four-band multispectral camera with a frame size of 57 × 57 $m{m}^{2}$ The instrument was designed as a monolithic unit, integrating the film magazine, body, and mount into one piece. The DGP-1-type multispectral camera, developed in 1976, was used for aerial photography in the Hami and Tengchong regions^30^. In 2016, Jia Ping et al. at CIOMP conducted a demonstration study on the application of large-scale UAV aerial remote sensing in disaster reduction. By equipping a comprehensive pod that integrates 13 types of sensors, they were able to acquire information from disaster scenes at a distance. They also carried out positioning experiments for natural disasters such as landslides, floods, and debris flows in Erhai County, Yunnan, utilizing a UAV-mounted integrated air, space, and ground disaster site information acquisition technology system to obtain real-time monitoring data from disaster sites^111^.

In the prevention and control of natural disasters and geological surveys, the importance of aerial surveying technology is becoming increasingly prominent. However, airborne cameras typically use general digital cameras, which have a small field of view, low resolution, and significant distortion. Direct use in surveying scenarios can lead to low efficiency and significant distortion.

To address the needs of surveying scenarios, Swiss Leica introduced the digital surveying camera ADS linear array series in 2000^112^, and Vexcel launched the UltraCAM series of products, which focused on geological surveys and other surveying needs since the beginning of the 21st century. These products can produce high-precision geographic information products for various scenes, including orthographic and oblique scenes.

Since 2012, the large field of view three-line array stereo camera, AMS-3000, developed by CIOMP, has further enhanced its application in geological surveys. This camera can obtain high-precision two-dimensional orthophotos and three-dimensional digital models, performing well in areas such as digital city construction and disaster relief^113,114^. A series of calibration methods for linear array cameras have also been proposed based on this camera^115^.

In addition, in the field of area array surveying cameras, Liu et al. designed a novel optical image motion compensation system for a tilted aplanatic secondary mirror (STATOS) optical system, reducing the sensitivity of the optical system’s image quality to image shift and significantly improving the MTF of the area array aerial surveying camera^116^.

In summary, the airborne optics of CIOMP started in 1958 and has long lagged behind those of developed countries in Europe and America. However, by the 2010s, in terms of various unit technologies, testing capabilities, and indicators of imaging systems developed, it had reached the international advanced level. It has played an important role in fields such as agriculture, disaster management, geology, and urban surveys. Furthermore, with the continuous expansion of airborne optical imaging technology and its applications, future developments in this field are likely to follow several key trends. These include intelligence, multimodality, high resolution, wide field of view and long distance.
